# Sociodemographic and behavioral factors associated with physical activity in Brazilian adolescents

**DOI:** 10.1186/1471-2458-14-485

**Published:** 2014-05-21

**Authors:** Leandro Fornias Machado de Rezende, Catarina Machado Azeredo, Daniela Silva Canella, Rafael Moreira Claro, Inês Rugani Ribeiro de Castro, Renata Bertazzi Levy, Olinda do Carmo Luiz

**Affiliations:** 1 Universidade de São Paulo - Faculdade de Medicina, Av. Dr. Arnaldo 455, 1° andar., São Paulo, São Paulo 01246-903, Brazil; 2 Universidade Federal de Uberlândia – Faculdade de Medicina, Av. Dr. Arnaldo 455, 1° andar., São Paulo, São Paulo 01246-903, Brazil; 3 Universidade de São Paulo - Faculdade de Saúde Pública, Av. Dr. Arnaldo, 715, São Paulo SP, Brazil; 4 Universidade Federal de Minas Gerais, Escola de Enfermagem, Av. Prof. Alfredo Balena, 190, Belo Horizonte, MG 01246904, Brazil; 5 Universidade do Estado do Rio de Janeiro. Instituto de Nutrição, Rua São Francisco Xavier, 524, 12° andar, Bloco E, sala 12007, Rio de Janeiro, Rio de Janeiro 20559-900, Brazil

**Keywords:** Motor activity, Physical activity, Sedentary lifestyle, Adolescent, Cross-sectional studies, Health surveys, Socioeconomic factors, Health behavior, Brazil

## Abstract

**Background:**

Physical activity in adolescents is associated with short- and long-term health benefits. Physical activity can occur in various domains and is influenced by a complex network of factors. The aims of this study are 1) to describe the physical activity of Brazilian adolescents in physical education classes, during leisure time, and during active commuting and 2) to investigate the socio-demographic and behavioral factors associated with physical activity.

**Methods:**

The representative sample included 109,104 Brazilian students in the final year of elementary school from 2,842 schools. The weekly frequency and duration of physical activity were assessed. A variety of socio-demographic and behavioral factors were studied. A multiple Poisson regression analysis was used to test for associations between physical activity and the socio-demographic and behavioral variables.

**Results:**

Most of the students (97.0%) engaged in physical activity in at least one of the domains studied, especially physical education at school (81.7%) and leisure time physical activity (67.5%). However, only 29% of the adolescents reached the recommended level of physical activity. Among the adolescents who reached the minimum recommended time for physical activity, the various domains contributed the following proportions to total physical activity: leisure time physical activity (PR 12.5; 95% CI 11.17-13.97), active commuting (PR 1.63; 95% CI 1.59-1.67), and physical education at school (PR 1.36; 95% CI 1.29-1.44). The weekly frequency of all activities was greater among boys than among girls. Moreover, nearly two-thirds (61.8%) of students spent more than two hours per day engaging in sedentary behaviors; the prevalence of sedentary behaviors was similar between boys and girls (59.0 and 64.5%, respectively).

Total level of physical activity, leisure time physical activity, and active commuting were associated with higher nutritional scores.

**Conclusions:**

Physical activity is important in any health promotion program. Therefore, it is necessary to invest in policies and interagency initiatives that promote all domains and to ensure that the general population helps determine the scope and design of such policies.

## Background

The importance of physical activity for health has been demonstrated in various studies [1,2]. During childhood and adolescence, the benefits offered by moderate-to-vigorous physical activity (MVPA) include lower cardiometabolic risk [3,4], less fat mass [3,5], and greater physical fitness (especially cardiorespiratory) [3]. In addition to these immediate results, physical activity in adolescence increases the probability of becoming an active and healthy adult [6] and lowers the risk of future health problems, such as fractures [7] and breast cancer [8]. Nevertheless, worldwide, fewer than 20% of adolescents aged 13 to 15 years practice physical activity at the recommended levels (more than 60 minutes of MVPA per day) [9].

Physical activity is determined by complex factors that vary widely between countries, and thus, it is important to research this topic [10]. Paradoxically, although 80% of the global population is located in low-and middle-income countries, only a small fraction of studies on the determinants of physical inactivity are conducted in these nations [10,11].

In Brazil, physical activity among adolescents has been studied in school, during sports participation, during leisure time, and during active commuting [12-14] However, few studies with representative data for the entire Brazilian population have investigated the role of socio-demographic and behavioral variables in determining the level of physical activity in adolescents [15]. The present study’s aims are as follows: 1) to describe the physical activity of Brazilian adolescents in various domains (physical education at school, during leisure time, and during active commuting) and 2) to investigate the socio-demographic and behavioral factors associated with physical activity.

## Methods

### Study population, sampling, and data collection

Data collected for the National Adolescent School-based Health Survey (Pesquisa Nacional de Saúde do Escolar–PeNSE), which was conducted between April and September 2012, were used in the present study. The aim of the PeNSE (2012) was to assess the risk and protective factors for health in a population of students in the final (ninth) year of elementary education at public and private schools throughout Brazil [16].

The geographic strata used for the 2012 PeNSE included the capital and other municipalities for each unit in the federation. The sample design was distinct for each stratum. In the capitals, cluster sampling was performed in two stages; the schools and the classes were the primary and secondary sampling units, respectively. In the remaining municipalities, three stages were used; the primary unit was the group of municipalities, the schools were the secondary unit, and the classes were the third unit. Of the 3,004 eligible schools, 162 were not selected because of the lack of ninth year elementary education classes, strikes, or refusal by the school board. Eighty-four percent (110,873) of the students routinely attended their classes. Among those students, 1,651 refused to participate, and 118 did not provide information on their gender and/or age. Those students were therefore excluded from the analyses. The final survey response rate was 83%. The sample was reweighted to represent the students who regularly attended classes. The present study used data from 109,104 students at 2,842 schools. Additional details about the PeNSE sampling process were described in the study report [16].

The collected data were recorded by the students themselves using an electronic structured questionnaire on a smartphone. The questionnaire was based on the version used in the *Global School-Based Student Health Survey*[17], the *Youth Risk Behavior Surveillance System*[18], and other national research projects [19-22]. The questionnaire was divided into 12 modules covering the following topics: socio-demographic variables, nutrition, body image, physical activity, smoking, use of alcohol and other drugs, oral health, sexual behavior, violence, accidents, safety, and self-reported anthropometric measurements.

### Analyzed variables and organization of the data

Physical activity was the main outcome of interest; the study examined the associations between physical activity and socio-demographic features, sedentary behavior, healthy nutrition, and the use of cigarettes, alcohol, and other drugs.

Physical activity was determined by measuring the weekly frequency and duration of various types of physical activity during the seven days preceding the study. The domains of physical activity included physical education at school, leisure time physical activity, and active commuting to and from school.

Physical activity in each domain was expressed as the percent distribution of weekly frequency. Additionally, the prevalence percentages of leisure time physical activity, active commuting (both defined as one or more weekdays), and physical education at school (defined as two or more weekdays, depending on the classes per week offered in most Brazilian schools) were estimated.

The total level of physical activity (LPA) was the sum of the weekly time spent active commuting, engaging in physical education at school, and performing sports and leisure activities. LPA was categorized as < 300 min/week or as ≥ 300 min/week, based on the level recommended by the World Health Organization [23].

The following socio-demographic variables were assessed: region (north, northeast, southeast, south, or midwest); municipality (capital or not); gender; age group (<13, 14–15, or > 16 years); race (white, black, yellow, brown, or indigenous); mother’s education level (incomplete elementary education, incomplete secondary education, incomplete higher education, or complete higher education); type of school (public or private); the presence of a vehicle in the household (car or motorcycle); and the work status of the adolescent (whether they had a job or business). Due to the significant proportion of missing values for the mother’s education level (17%, n = 18,527), multiple imputation was applied using the *multiple imputation by chained equation* method based on predictive variables such as gender, the father’s education level, and variables related to the ownership of goods and access to services. Multiple imputation was performed 10 times, and the Monte Carlo error analysis showed good statistical reproducibility of the results [24].

The presence of a vehicle in the household was assessed because of its potential effect on active commuting. The following behavioral variables were used: sedentary behavior, healthy nutrition, and the use of cigarettes, alcohol, and drugs in the previous 30 days. Sedentary behavior in adolescents was defined according to the number of hours spent in front of a television, computer, or video game or engaging in seated activities with friends (excluding time at school) on a regular weekday. The total time invested in such activities was classified as < 120 min/day or ≥ 120 min/day [25]. The use of cigarettes, alcohol, and drugs was defined as the reported consumption of these substances at least once within the previous 30 days. A score based on healthy foods and preparation methods (beans, raw vegetables, cooked vegetables, fresh fruits, and milk) and unhealthy foods and preparation methods (soft drinks, candies, cookies, salty snacks, fried foods, cured sausages) was developed to assess dietary intake. The score for each item was determined by the weekly intake frequency. The scores for healthy items ranged from 0 (never) to 7 (daily); the scores for unhealthy items ranged from 0 (daily) to 7 (never). Therefore, the total score could range from 0 to 77. Higher scores indicate better nutritional quality. Cronbach’s alpha was used to assess the internal consistency (0.7) of the variables for the nutritional score. The correlation between items was also assessed and ranged from −0.0004 to 0.40 [26]. The nutritional score was analyzed as a continuous variable, and the mean and standard error (SE) are presented to describe the sample. Subsequently, the score was categorized into tertiles for the multiple regression analysis.

### Statistical analysis

The study’s variables of interest were expressed as absolute and relative frequencies for the entire population and by gender. The weekly frequency of physical activity in the various domains (physical education at school, leisure time physical activity, active commuting to and from school, and LPA) was also estimated for the entire population and by gender.

A Poisson regression in crude analysis was used to determine the variables that best predicts physical activity in each domain. The selection of the variables for the multiple regression was determined by a mathematical criteria p < 0.20 in at least one of the domains. The prevalence, adjusted prevalence ratio (estimated from multiple Poisson regression models used to estimate the prevalence ratio), and respective confidence interval (95% CI) for each physical activity domain (> 2 days/week of physical education at school, > 1 day/week of active commuting, > 1 day/week of sports and leisure activities without an instructor, ≥ 300 min/week for LPA) were estimated for the entire population and for the strata defined by socio-demographic and behavioral variables. The sample design was taken into consideration for all descriptive analyses. The analyses were performed using Stata 12.1 software (StataCorpo LP, College Station, TX, USA).

### Ethical considerations

The PeNSE was approved by the National Commission for Research Ethics (Comissão Nacional de Ética em Pesquisa–CONEP; under protocol number 16,805), and access to the study database is freely available through the IBGE website without identifying information for the participants.

## Results

More than two-thirds of the students studied resided in south-central Brazil (66.7%), and slightly more than one-fifth (22.4%) resided in state capitals. Most students were aged 14 to 15 years (63.9%), attended public school (82.8%), and self-identified as black or brown (55.6%). Less than one-sixth of the students (13.2%) reported working. Nearly half of the students (39.0%) were the children of mothers who did not complete elementary school. Slightly more than one-tenth of the students (12.5%) had mothers with higher education. Approximately two-thirds of the students came from families with at least one vehicle (63.8%). In the 30 days preceding the study, 5.1% of the adolescents smoked, 26.1% drank alcohol, and 2.4% used drugs. The mean nutritional score for the students was 42.50 (SE = 0.22) (Table 1).

**Table 1:** Socio-demographic features, sedentary behavior, and physical activity in various domains for adolescents, by gender-Brazil, 2012

**Variable**	**Total**		**Boys**		**Girls**	
	**N**	**%**	**N**	**%**	**N**	**%**
Region*						
North	22,774	8.0	10,574	7.6	12,200	8.3
Northeast	31,301	25.3	14,355	23.4	16,946	27.0
Southeast	19,660	44.3	9,685	46.2	9,975	42.6
South	14,878	14.6	7,304	14.8	7,574	14.3
Midwest	20,491	7.9	10,097	8.0	10,394	7.8
Municipality*						
Capital	61,145	22.4	29,393	23.0	31,752	21.8
Non-capital	47,959	77.6	22,622	77.0	25,337	78.2
Age*						
≤ 13	22,443	22.9	9,148	19.8	13,295	25.8
14 – 15	72,005	63.9	34,471	64.4	37,534	63.4
≥ 16	14,656	13.2	8,396	15.8	6,260	10.8
Race*						
White	37,674	36.8	51,180	38.7	48,734	35
Black	14,513	13.4	23,549	15.5	17,824	11.4
Yellow	4,821	4.1	5,640	3.7	7,341	4.4
Brown	48,237	42.2	57,760	38.5	71,877	45.7
Indigenous	3,790	3.5	5,130	3.6	5,000	3.5
Type of school*						
Private	22,504	17.2	11,066	17.7	11,438	16.7
Public	86,600	82.8	10,949	82.3	45,651	83.3
Work*						
Yes	14,318	13.2	9,101	17.4	5,217	9.2
No	94,666	86.8	42,853	82.6	51,813	90.8
Mother’s education level*						
Incomplete elementary	37,629	39	16,840	36.7	20,838	36.7
Incomplete secondary	18,978	17.8	9,019	17.9	9,907	17.6
Complete secondary	35,448	30.7	17,413	31.6	18,048	29.8
Complete higher	17,015	12.5	8,863	13.7	8142	11.4
Has automobile/motorcycle*						
Yes	72,655	63.8	35,709	66.2	36,946	61.8
No	36,252	36.2	16,216	33.8	20,036	38.2
**Cigarettes**						
Yes	5,748	5.1	2,937	5.1	2,811	5.0
No	103,078	94.9	48,928	94.9	54,150	95.0
Alcohol*						
Yes	27,763	26.1	12,759	25.2	15,004	26.9
No	80,905	73.9	39,001	74.8	40,904	73.1
Drugs*						
Yes	2,842	2.4	1,668	2.8	1,174	2.0
No	105,911	97.6	50,154	97.2	55,757	98.0
**Nutritional score**	42.50	0.22	43.68	0.16	41.43	0.29

*p < 0,01.

Most of the students (97.0%) engaged in physical activity in at least one of the domains studied, especially physical education at school (81.7%) and leisure time physical activity (67.5%). The weekly frequency of all activities was greater among boys than among girls (Table 2). Only 29% reached the recommended level (> 300 min/week) of physical activity. Boys reached the recommended levels more often than girls (38.6% vs. 20%, respectively). Moreover, nearly two-thirds (61.8%) of students spent more than two hours per day engaging in sedentary behaviors; the prevalence of sedentary behaviors was similar between boys and girls (59.0 and 64.5%, respectively).

**Table 2:** Proportion of weekly physical activity in the various domains, LPA, and sedentary behavior among adolescents-Brazil, 2012

**Variables**	**Weekly frequency (%)**						**p value**
	**0**	**1**	**2**	**3**	**4**	**≥ 5 days**	
**Physical education at school**							< 0,001
Boys	16.8	42.6	28.1	7.1	2.1	3.2	
Girls	19.7	43.5	26.5	6.3	1.7	2.3	
Total	18.3	43.1	27.3	6.7	1.9	2.7	
**Leisure time physical activity**							< 0,001
Boys	23.4	12.8	12.6	11.3	7.3	32.7	
Girls	41.0	14.8	11.7	9.1	4.9	18.5	
Total	32.6	13.9	12.1	10.1	6.1	25.3	
**Physical activity while commuting**							< 0,001
Boys	36.1	5.3	2.9	2.7	2.1	50.9	
Girls	39.8	4.8	2.6	2.0	1.6	49.2	
Total	38.0	5.0	2.8	2.3	1.8	49.9	
Total physical activity*							< 0,001
Boys	2.3	5.0	7.0	5.6	5.5	74.6	
Girls	4.2	9.6	9.0	6.3	5.5	65.5	
Total	3.3	7.4	8.0	6.0	5.5	69.8	
**60 minutes/day**							< 0,001
Boys	14.4	18	16.3	14.2	9.2	27.9	
Girls	33.9	21.8	15.5	10.2	5.5	13.1	
Total	24.6	20	15.8	12.1	7.3	20.2	

*Includes all domains.

The results of the adjusted multiple regression models revealed the set of factors associated with each domain of physical activity. The prevalence of physical education at school was higher among the students who resided in capital municipalities, students aged 15 years or younger, white students, those who reported working, and those from a family with a vehicle (Table 3).

**Table 3:** Adjusted* Poisson regression analysis of the association between physical activity domains and socio-demographic features - Brazil, 2012

**Variable**	Physical education at school*			Leisure time physical activity**			Active commuting**			Level of physical activity***		
	**%**	**PR**	**95% CI**	**%**	**PR**	**95% CI**	**%**	**PR**	**95% CI**	**%**	**PR**	**95% CI**
**Region**												
North	35.2	1		70.5	1		63.3	1		28.3	1	
Northeast	27.4	0.84	0.39-1.80	66	**0.95**	**0.93-0.97**	57.5	0.93	0.85-1.00	24.3	0.96	0.91-1.01
Southeast	34.2	1.03	0.42-2.53	66.8	**0.93**	**0.91-0.94**	62.3	0.98	0.90-1.06	29.2	1.03	0.94-1.13
South	69.7	2.05	0.95-4.43	70.3	**0.93**	**0.90-0.96**	65.3	1.02	0.91-1.13	35.1	**1.08**	**1.02-1.14**
Midwest	46.8	1.30	0.62-2.72	68	**0.93**	**0.91-0.95**	66.3	1.03	0.95-1.12	31.7	1.04	0.98-1.11
**City**												
Capital	49.4	1		67.2	1		61.8	1		31.7	1	
Non-capital	35.6	**0.65**	**0.43-0.98**	67.6	**1.03**	**1.02-1.05**	61.9	1.00	0.94-1.06	28.2	0.96	0.92-1.01
**Gender**												
Female	36.8	1		59.1	1		60.1	1		20.1	1	
Male	40.7	1.02	1.00-1.04	76.7	**1.16**	**1.15-1.18**	63.9	1.01	0.99-1.03	38.6	**1.46**	**1.43-1.50**
**Age**												
≤ 13	39.9	1		68.9	1		62.5	1		28.5	1	
14-15	38.8	0.97	0.93-1.01	67.6	**0.97**	**0.95-0.99**	62.7	0.99	0.98-1.00	29.6	1.03	1.00-1.06
≥ 16	36.5	**0.95**	**0.91-0.98**	64.7	**0.93**	**0.90-0.95**	59.9	**0.90**	**0.88-0.91**	26.7	0.99	0.96-1.02
**Race**												
White	43.5	1		68.5	1		60.8	1		30.9	1	
Black	37.5	**0.95**	**0.91-0.99**	66.7	0.99	0.97-1.02	61.9	1.01	1.00-1.03	29	1.00	0.97-1.03
Yellow	35	**0.89**	**0.85-0.94**	66.5	1.00	0.97-1.02	61.5	**1.03**	**1.01-1.04**	28.6	1.01	0.97-1.04
Brown	35.6	**0.93**	**0.91-0.95**	66.9	1.02	1.00-1.03	62.9	**1.04**	**1.02-1.05**	27.2	1.00	0.98-1.02
Indigenous	34.7	**0.90**	**0.85-0.95**	69.3	1.03	0.95-1.11	61.2	1.00	0.99-1.03	30.1	1.06	0.93-1.21
**Type of school**												
Private	30.6	1		72.7	1		53.8	1		34.5	1	
Public	40.4	1.41	0.99-2.01	66.5	**0.95**	**0.94-0.96**	63.6	**1.20**	**0.99-1.45**	27.8	**0.86**	**0.78-0.94**
**Work**												
Yes	43.5	1		72.6	1		65.4	1		35.9	1	
No	38	**0.96**	**0.93-0.99**	66.7	0.98	0.97-1.00	61.3	**0.96**	**0.94-0.98**	27.9	**0.92**	**0.91-0.94**
**Mother’s education level**												
Incomplete elementary	38.9	1		64.8	1		60.6	1		24.9	1	
Incomplete secondary	38.4	**0.94**	**0.90-0.98**	67.7	1.02	1.00-1.03	64.7	**1.05**	**1.03-1.08**	28.6	**1.05**	**1.02-1.09**
Complete secondary	37.8	**0.92**	**0.90-0.95**	68.7	1.01	0.99-1.02	64	**1.05**	**1.02-1.08**	31.2	**1.11**	**1.06-1.16**
Complete higher	41	1.00	0.95-1.06	72.9	1.02	0.99-1.04	56.6	0.96	0.88-1.04	36.4	**1.20**	**1.14-1.26**
**Has automobile/motorcycle**												
No	34.7	1		63.8	1		64	1		25.5	1	
Yes	40.9	**1.09**	**1.04-1.15**	69.6	**1.04**	**1.03-1.05**	60.7	**0.94**	**0.93-0.96**	30.8	1.03	0.99-1.07

Adjusted: sedentary behavior; nutritional score; use of cigarettes, alcohol and drugs; other physical activity domains and variables in the table.

Crude PR might be obtained by dividing the prevalence between strata.

*Involved at least 2 days per week.

**Involved at least 1 day per week.

***At least 300 minutes of physical activity per week.

Bold data reflect statistical significance (p < .05).

With regard to leisure time physical activity, the prevalence was greater among adolescents from the north region, those residing in rural municipalities, males, those younger than 13 years, those attending private schools, and those who reported having a vehicle in the household (Table 3).

The prevalence of active commuting was higher among students of yellow and brown race, those aged 15 years or younger, those who worked, those whose mothers had a secondary education (complete or incomplete), and those from families without a vehicle (Table 3).

Among the adolescents who reached the minimum recommended time for physical activity, the various domains contributed the following proportions to total physical activity: leisure time physical activity (PR 12.5; 95% CI 11.17-13.97), active commuting (PR 1.63; 95% CI 1.59-1.67), and physical education at school (PR 1.36; 95% CI 1.29-1.44).

Leisure time physical activity, active commuting, and LPA were associated with higher nutritional scores. However, physical education at school was associated with smoking at least once during the previous 30 days. Similarly, greater levels of physical activity were associated with the use of alcohol and drugs at least once during the previous 30 days (Table 4).

**Table 4:** Adjusted* Poisson regression analysis of the association between physical activity domains and lifestyle habits-Brazil, 2012

**Variables**	PE at school*			Leisure PA **			Active commuting**			LPA***		
	**%**	**PR**	**95% CI**	**%**	**PR**	**95% CI**	**%**	**PR**	**95% CI**	**%**	**PR**	**95% CI**
**Total**	38.7		28.6-48.7	67.5		66.7-68.3	61.9		59.9-64.0	28.8		28.1-29.5
**Physical education at school**												
No	-	-	-	65.5	1		61.2	1		24.1	1	
Yes	-	-	-	70.6	1.00	0.99-1.02	62.9	**0.97**	**0.95-0.98**	36.6	**1.36**	**1.29-1.44**
**Leisure time physical activity**												
No	35	1		-	-	-	56	1		0.3	1	
Yes	40	1.00	0.98-1.03	-	-	-	64.8	**1.04**	**1.02-1.06**	41.5	**12.5**	**11.17-13.97**
**Active commuting**												
No	37.6	1		62.5	1		-	-	-	19	1	
Yes	39.4	**0.95**	**0.93-0.98**	70.6	**1.03**	**1.02-1.04**	-	-	-	35	**1.63**	**1.59-1.67**
**Level of physical activity**												
Inactive	34.6	1		55.6	1		56.6	1		-	-	-
Active	48.9	**1.33**	**1.22-1.45**	96.8	**1.66**	**1.65-1.67**	74.9	**1.30**	**1.28-1.33**	-	-	-
**Sedentary behavior**												
< 2 hours/day	37.8	1		68.4	1		58.2	1		28	1	
> 2 hours/day	39.3	0.99	0.94-1.05	66.7	**0.98**	**0.97-0.99**	64.2	**1.11**	**1.09-1.13**	29.5	**1.04**	**1.01-1.06**
**Diet score**												
1st tertile	38.2	1		63.5	1		39.2	1		26.7	1	
2nd tertile	38.2	1.00	0.97-1.02	67.2	**1.04**	**1.03-1.06**	38.5	1.01	0.98-1.03	26.7	**0.94**	**0.92-0.97**
3rd tertile	39.5	1.00	0.97-1.03	72	**1.07**	**1.06-1.08**	36.3	**1.04**	**1.01-1.07**	33	**1.09**	**1.07-1.12**
**Cigarettes**												
Yes	49.9	1		69	1		61.7	1		33.3	1	
No	**38.1**	**0.93**	**0.89-0.96**	67.4	0.99	0.97-1.02	61.9	1.05	0.99-1.10	28.7	0.99	0.95-1.05
**Alcohol**												
Yes	42.9	1		68.8	1		62.1	1		31.5	1	
No	37.2	**0.96**	**0.93-0.99**	67	**0.97**	**0.96-0.99**	61.9	1.01	0.99-1.02	28	**0.94**	**0.92-0.95**
**Drugs**												
Yes	53.2	1		72	1		65.4	1		40.8	1	
No	38.3	**0.94**	**0.89-0.99**	67.4	1.02	0.99-1.04	61.8	0.98	0.93-1.00	28.6	**0.93**	**0.89-0.97**

Adjusted: region; city; gender; age; race; mother’s education level; type of school; presence of vehicle in the household; work; other physical activity domains and variables in the table.

Crude PR might be obtained by dividing the prevalence between strata.

*Involved at least 2 days per week.

**Involved at least 1 day per week.

***At least 300 minutes of physical activity per week.

Bold data reflect statistical significance (p < .05).

## Discussion

This study was the first Brazilian study to investigate the association between physical activity in various domains with socio-demographic and behavioral variables among adolescents using data from the nationally representative PeNSE 2012. The results of the present study indicate that only 29% of adolescents reach the recommended 300 minutes of physical activity per week, despite the high prevalence of physical activity. In 2009, the prevalence of physical activity in adolescents was 43, 1% (56, 2% in boys and 31, 3% in girls), however it is important to highlight that the coverage of the research and the physical activity questionnaire (commute and leisure time domains) has been changed for PeNSE 2012. In other Latin American countries such as Argentina, Chile and Venezuela around 10% of the adolescents are physically active (more than 60 min per day of moderate to vigorous physical activity), while in Uruguay, Peru, Ecuador, Colombia, Guyana and Suriname this proportion increases to 20% [9].

Brazil is a country with a continental dimension with a persisting social, cultural and economic heterogeneity. Programs and policies that promote physical activity should take into account differences between geographic locations. We found no difference for the prevalence of physical education at school among the regions in the adjusted models. As the country has a national policy for this matter, the differences verified (unadjusted) are possibly relate to the lack of infrastructure (such as sports goods and adequate physical space). This is probably the case in the North and Northeast regions (less developed regions of the country) and may by the case in the Southeast region (where the biggest share of the public schools are located, increasing the complexity of its administration). Leisure time physical activity was more prevalent in the North region, probably due to cultural factors and urban environments that allow (or promote) this domain of activity, situation less often verified in the regions South, Southeast and Midwest. However, accurately understanding such a phenomena becomes difficult facing rare/none publications in physical activity field concerning specifically the situation in each region and the lack of information about possible determinants of the disparities in PeNSE. Thus, studies, and even the surveillance system, designed specifically to understand the correlates/determinants of physical activity domains across the Brazilian regions are necessary”.

Additionally, students in rural municipalities participated in less physical education at school; however, these locations had a greater proportion of leisure time physical activity. Socioeconomic class is another aspect that should be considered. Higher education levels were associated with a greater probability of reaching the recommended levels of physical activity. A similar finding was reported by another study [27].

As in the other domains, there was less participation in physical education classes among older students, corroborating the results of prior studies [12,28]. However, this age-related difference did not hold for meeting the recommended level of physical activity. The prevalence of physical education at school and active commuting was similar between the genders. Women are less involved in physical activity at all ages [12]. Therefore, proper planning of physical education at school and incentives for active commuting could be good strategies for getting women to reach the minimum recommended weekly levels of physical activity.

Despite high adherence among adolescents (82%), physical education at school only increased the probability of achieving the recommended levels by 36%. This finding can be explained by the low frequency and short duration of physical education classes. In Brazil, although law requires physical education classes, the minimum number of classes per week is not specified [29,30]. They are generally offered twice a week and last 50 minutes, for a total of 100 minutes per week; this time is one-third of the recommended level for the age group. In addition, studies have shown that students are engaged in moderate-to-vigorous physical activity (objective measurements of MVPA) for only 20-47% of the physical education class time [31-33]. Therefore, increasing the frequency and quality of physical education classes could improve the health of adolescents and promote activity in adulthood [34]. School is a suitable environment for developing active behavior: it is relatively safe, the required space and equipment are available, and appropriate professional guidance is offered. Therefore, school is a strategic location for reducing levels of physical inactivity in infants and adolescents. In addition, future studies could investigate environmental, sociocultural, and different physical education classes approach that are strongly related to a better adhesion to the practice in this domain.

Among the domains we studied, leisure time physical activity was the most important (PR 12.5; 95% CI 11.17-13.97) for achieving the recommended level of physical activity. This finding strongly suggests that adolescents who reach the recommended minimum of 300 minutes of physical activity per week do so more because of leisure time physical activity than because of physical education at school or active commuting. Leisure time physical activity is the main way that adolescents incorporate physical activity into their daily lives, reflecting the preferences of the study population. Therefore, strategies that promote physical activity by focusing on leisure time [35] could see better adherence rates, especially for groups of people who are just beginning to engage in physical activity (i.e., public school students, girls, and the children of mothers with little education). Studies have shown that interventions in public spaces, with easily accessible facilities and proper guidance, affect individual choices to engage in physical activity [10,36-38]. Providing appropriate public spaces lowers the physical, psychological, and financial barriers to leisure time physical activity [10,37]. Conducting programs at plazas, parks, and beaches, or even along avenues or in places near homes, encourages greater participation by the population [36,39]. For example, an ongoing community-base program, called *Academia da Cidade*, has showed to be an effective public health strategy to increase leisure-time physical activity in Brazilian urban settings (Recife) by providing PA classes in the community [40,41]. In contrast, the perception of a lack of safety due to urban violence and dark areas could act as a significant barrier to engaging in physical activities in such spaces [42]. Additionally, Parra et al. study has suggested that this is a complex relationship once different environmental characteristics in Curitiba are associated with different physical activity outcomes [43].

Active commuting increased the probability of adolescents achieving the minimum recommended levels of physical activity by 63%. In total, 62% of the adolescents commuted at least one day per week, and 50% commuted five or more days per week. This type of physical activity was more common among students younger than 15 years, those whose mothers had secondary education, those who lived in a house without a vehicle, and adolescents who worked. A similar finding was reported in another study in which active commuting to school was associated with lower socioeconomic class [44]. Active commuting is an important domain for reaching the recommended levels of physical activity. Moreover, it is easy and inexpensive and does not require much additional time in the daily routine. Commuting is a good way to engage in physical activity without making large changes to the daily routine; however, the major modifiable barriers related to the city (the distance between the starting point and the destination, traffic-related hazards, and safety concerns) and the school (an appropriate and safe location for parking bicycles, locker rooms with showers) should be addressed in interventions to promote active commuting among adolescents [45].

Despite the association between physical activity and other health behaviors, we found that leisure time physical activity, active commuting, and LPA were associated with higher nutritional scores. However, physical education at school was associated with smoking at least once during the previous 30 days. Similarly, greater levels of physical activity were associated with the use of alcohol and drugs at least once during the previous 30 days (Table 4). Due to the small magnitude of the relationship, we strongly believe that residual confounding (such as the lack of more accurate information about economic status) might be the cause of this situation. As previously reported by some studies [10,12], a clusterization of healthy behaviors was expected (as was one, complementary, for the unhealthy ones). An example of this includes the association between physical activity (leisure, commuting and total physical activity) and healthy diet score presented among our results.

The main limitation of this study was that physical activity was measured with a questionnaire and not with a direct method, such as an accelerometer. Although there is no agreement between continuous scores from questionnaires and those from accelerometers, the classification of participants into physical activity groups showed moderate to high agreement [27]. This agreement justifies the use of questionnaires for representative studies in large populations, such as the PeNSE. Additionally, a prior study that assessed students in the penultimate year of elementary school using a questionnaire and focus groups showed that the students were able to understand the questions and answers, were able to answer questions involving the selection of categories and frequencies, and left few questions blank [22]. The use of questionnaires was further supported by a validation study for the physical activity indicators used in the PeNSE, which indicated that the indicators performed well [46].

With regard to the completeness of the responses, most of the questionnaire items had response rates above 95%. Only the item about the mother’s education level had a higher (17%) non-response rate. Therefore, multiple imputation was used, and the expansion factors used for the analyses were recalculated considering the losses in coverage, which improved the results from multiple analyses. Imputation of missing data is always recommended because it considerably increases the reliability and power of the analyses [47].

With regard to external validity, the PeNSE involved a representative sample of Brazilian students from public and private schools. Brazil has a high coverage rate for elementary education: 88% of adolescents aged 15 to 19 years attend school [48]. Therefore, it is reasonable to assume that the same behaviors observed in the present study would be observed in the entire population of Brazilian adolescents.

## Conclusion

In conclusion, we found that different physical activity domains are associated with specific socio-demographic and health behaviors. Physical education at school was higher among the students who resided in capital municipalities, students aged 15 years or younger, white students, those who reported working, and those from a family with a vehicle. Leisure time physical activity was greater among adolescents from the north region, those residing in rural municipalities, males, those younger than 13 years, those attending private schools, and those who reported having a vehicle in the household. Active commuting was higher among students of yellow and brown race, those aged 15 years or younger, those who worked and whose mothers had a secondary education, and those from families without a vehicle. In this sense, physical activity programs should take into account those specificities.

Physical activity is an important component of any health promotion program. It is still necessary to invest in policies that promote physical activity in all domains and to encourage interagency actions that focus on health, education, sports, the environment, safety, transportation, and social communication. Such actions will ensure that the population participates in determining the scope and design of these policies.

## Abbreviations

MVPA: Moderate to vigorous physical activity; PeNSE: Pesquisa Nacional de Saúde do Escolar; LPA: Level of Physical Activity; CONEP: Comissão Nacional de Ética em Pesquisa; PR: Prevalence Ratio; CI: Confidence Interval; SE: Standard error.

## Competing interests

The authors declare that they have no competing interests.

## Authors’ contributions

LFMR, CMA, DSC, RBL, and OCL were involved in the concept and study design. LFMR, CMA, DSC conducted data analysis. LFMR and OCL drafted the manuscript. OCL, RMC, IRRC, and RBL critically revised the manuscript. All authors have read and approved the final version being submitted to the journal.

## Pre-publication history

The pre-publication history for this paper can be accessed here:

http://www.biomedcentral.com/1471-2458/14/485/prepub
